# Oral Health and “Modern” Digestive Diseases: Pathophysiologic and Etiologic Factors

**DOI:** 10.3390/biomedicines12081854

**Published:** 2024-08-15

**Authors:** Mihaela Rotaru, Ana-Maria Singeap, Alin Ciobica, Laura Huiban, Carol Stanciu, Laura Romila, Vasile Burlui, Ioannis Mavroudis, Anca Trifan

**Affiliations:** 1 Department of Biology, Faculty of Biology, “Alexandru Ioan Cuza” University of Iasi, Bd. Carol I No. 20A, 700505 Iasi, Romania; rotmih97@gmail.com (M.R.); alin.ciobica@uaic.ro (A.C.); 2 Department of Gastroenterology, Faculty of Medicine, “Grigore T. Popa” University of Medicine and Pharmacy, Universitatii Street No. 16, 700115 Iasi, Romania; huiban.laura@yahoo.com (L.H.); stanciucarol@yahoo.com (C.S.); ancatrifan@yahoo.com (A.T.); 3 Institute of Gastroenterology and Hepatology, “St. Spiridon” Emergency County Hospital, Bd. Independentei No. 1, 700111 Iasi, Romania; 4 CENEMED Platform for Interdisciplinary Research, “Grigore T. Popa” University of Medicine and Pharmacy, Universitatii Street No. 16, 700115 Iasi, Romania; 5 Centre of Biomedical Research, Romanian Academy, Bd. Carol I No. 8, 700506 Iasi, Romania; 6 Academy of Romanian Scientists, Splaiul Independentei Street No. 54, 050094 Bucharest, Romania; 7 “Ioan Haulica” Institute, Apollonia University, Pacurari Street No. 11, 700511 Iasi, Romania; vburlui@gmail.com; 8 Department of Neuroscience, Leeds Teaching Hospitals, NHS Trust, Leeds LS2 9JT, UK; i.mavroudis@nhs.net; 9 Third Department of Neurology, Aristotle University of Thessaloniki, 54124 Thessaloniki, Greece

**Keywords:** irritable bowel syndrome, intestinal dysbiosis, inflammatory bowel disease, oral diseases, small intestinal bacterial overgrowth, non-alcoholic fatty liver disease

## Abstract

In the contemporary era of medicine, exploring the complexity of the human body and its intricate interactions has become a central concern for health researchers. The main purpose of this article is to summarize the current understanding of relevant pathophysiological factors such as chronic inflammation, dysbiosis (microbial imbalance), and metabolic disorders, as well as etiological factors including dietary habits, lifestyle choices, obesity, metabolic syndrome, and genetic predispositions, as well as to emphasize potential avenues for upcoming studies and their medical significance. Additionally, this article aims to assess the potential impact of integrated treatment approaches on patient outcomes, emphasizing the need for interdisciplinary collaboration between gastroenterologists, dentists, and other healthcare professionals to develop comprehensive care plans that address both oral and digestive health issues simultaneously. Among the branches with a significant impact on general well-being are oral cavity health and digestive diseases, which have been the subject of intensive research in recent decades. In this context, analysis of the current state of knowledge on oral cavity disorders in relation to “modern” digestive diseases such as non-alcoholic fatty liver disease (NAFLD), small intestinal bacterial overgrowth (SIBO), inflammatory bowel disease (IBD), and irritable bowel syndrome (IBS) becomes essential for a deeper understanding of the interconnections between oral and digestive health. The temporal overlap or succession, whether preceding or following, of oral manifestations and digestive disorders should be taken seriously by both gastroenterologists and dentists to facilitate early diagnosis and explain to patients the correlation between these two body systems. In summary, this article underscores the importance of understanding the intricate relationship between oral and digestive health, advocating for interdisciplinary approaches to improve patient outcomes and guide future research.

## 1. Introduction

Oral health is vital to the overall health and well-being of all people. Oral health encompasses more than just the aesthetics and condition of teeth; it involves the well-being of the oral mucosa, periodontium, tongue, and lips, which means the overall appearance of the face [1]. Oral health significantly impacts an individual’s dental function, social interactions, and overall well-being. Moreover, oral diseases are among the most prevalent health conditions globally [2]. The most common and significant oral diseases globally are dental caries, periodontal disease, tooth loss, and cancers affecting the lips and oral cavity. The number of missing teeth can serve as a barometer of overall oral health, reflecting the cumulative inflammatory and infectious status of oral health. It is widely recognized as a primary indicator of general oral health conditions [3]. The prevalence of oral disease has adverse health effects, including body image problems, sleeplessness, social withdrawal, physical suffering, distress, apprehension, worry, and restricted abilities [4]. Improper diet, involving increased consumption of sweet foods and drinks, smoking, alcohol consumption, and poor oral hygiene are the most important factors influencing the occurrence of various oral disorders [5]. The prevalence of dental caries is maintained at a high level in Romania, and this has been demonstrated in population surveys. In the first study, the presence of dental disease was highlighted in 687 children (85.3%) out of 809 with an average age of 6 years [6]. In another study conducted among teenagers, the prevalence of dental caries was 95.9% in the group of girls and 95.2% in the group of boys [7].

The oral cavity, which possesses anatomical and functional diversity, serves not only as a gateway for food but also as a complex ecosystem of microorganisms. On the other hand, “modern” digestive diseases such as non-alcoholic fatty liver disease (NAFLD), small intestinal bacterial overgrowth (SIBO), irritable bowel syndrome (IBS), and inflammatory bowel disease (IBD) have become increasingly prevalent, having a significant impact on quality of life and inducing challenges for healthcare professionals [8].

Over the past four decades, extensive research has established a strong connection between oral health and the rest of the body, identifying over 50 systemic conditions with gum disease and periodontal inflammation [9,10,11]. 

Digestive health is fundamental to overall well-being, encompassing the proper functioning of the gastrointestinal tract and the maintenance of a balanced gut microbiota. The human gut microbiome has become a focal point of intensive research in recent years, with our understanding of its resident organisms and their potential capacity rapidly growing [12]. The digestive system oversees the breakdown and nutrient assimilation, playing a critical role in sustaining bodily functions and supporting immune health. However, various factors can disrupt digestive health, leading to a range of disorders that impact quality of life.

Microbiota manipulation techniques are pivotal in both medical practice and research settings to modify the composition and function of the gut microbiome, thereby impacting host health. These techniques encompass a variety of strategies, including dietary interventions, probiotic and prebiotic supplementation, and fecal microbiota transplantation (FMT). 

Probiotic bacteria, present naturally within the human digestive system, play a crucial role in maintaining and enhancing the health of the host. These beneficial microorganisms contribute to a balanced gut microbiota, which is indispensable for optimal gastrointestinal function. The therapeutic potential of probiotics has been extensively studied, and their benefits are linked to their ability to alter the gut microbial composition and function [13,14]. Dietary interventions involve altering macronutrient intake to promote beneficial microbial growth. Probiotics, live microorganisms that confer health benefits, and prebiotics, non-digestible food components that stimulate growth or activity of beneficial bacteria, are commonly used to enhance gut health. FMT, the transfer of stool from a healthy donor to the gastrointestinal tract of a patient, has been particularly successful in treating *Clostridium difficile* infections and is being explored for other conditions such as inflammatory bowel disease and metabolic disorders. Advanced molecular techniques like metagenomics, metabolomics, and culturomics are increasingly utilized to identify and cultivate novel beneficial microbes, providing a deeper understanding of microbiota–host interactions and opening new avenues for therapeutic interventions [15,16].

Recent clinical trials are actively exploring the modulation of the gut microbiota and its impact on diseases along the gut–oral axis, including IBS, NAFLD, and SIBO. These trials aim to investigate the therapeutic potential of microbiota modulation strategies such as prebiotics, synbiotics, probiotics, and FMT. For instance, in IBS, studies are assessing the effectiveness of probiotics and FMT in alleviating symptoms and improving quality of life [17]. In NAFLD, clinical trials are focusing on the impact of gut microbiota on liver fat accumulation and inflammation. Recent clinical trials are actively exploring interventions like probiotics to manage these conditions, as presented in Table 1 [18]. Similarly, for SIBO, trials are evaluating the safety and efficacy of FMT. 

Irritable bowel syndrome (IBS) is a functional gastrointestinal condition with an unknown cause [19], with some authors considering the onset of symptoms to be related to disturbances occurring between the gut–brain axis [20], as patients with IBS often present with neurological dysfunctions such as anxiety, depression, and other symptoms. Other authors suggest the commitment of the inflammatory response in the onset of IBS as a triggering mechanism for gastrointestinal disorder [21]. Clinical studies and animal models have indicated a moderate involvement of oxidative stress in IBS, characterized by alterations in key indicators of antioxidant capacity and lipid peroxidation [22]. It is considered a major disorder associated with impaired quality of life, being accompanied and characterized by abdominal pain, altered bowel movements, and bloating, without significant damage to the intestinal mucosa. In a large-scale multicountry study, the prevalence of IBS was found to be 10.1% according to the Rome III criteria and 4.1% according to the Rome IV criteria [23]. 

The literature documents a link between inflammatory bowel disease (IBD) and an increased risk of developing inflammatory periodontal disease. It has been found that patients with IBD are approximately two and a half times more prone to developing periodontal disease. The results of the study by Koutsochristou et al. [24] exhibited higher rates of tooth decay and periodontal disease compared to their healthy peers, despite comparable oral hygiene indicators to those of the control group. Surprisingly, Grössner-Schreiber et al. [25] did not observe significant differences in caries’ incidence and periodontal status indices between IBD patients and healthy subjects. Similarly, in their study, Piras et al. demonstrated a higher prevalence of larger periapical lesions, despite no significant differences in caries rates compared to the control group, particularly evident in IBD patients, especially those undergoing immunomodulatory therapy [26].

NAFLD is a condition characterized by a range of liver abnormalities, progressing from simple fat accumulation (hepatic steatosis) to liver inflammation (steatohepatitis), fibrosis, cirrhosis, and even hepatocellular carcinoma. It appears in the absence of other specific causes of liver injury, such as alcohol consumption, viral B or C hepatitis, or hepatotoxic drugs [27]. The pathogenesis of NAFLD is strongly linked to metabolic conditions, including obesity, dyslipidemia, hypertension, hyperglycemia (most often associated with the development of type 2 diabetes), and persistent liver function test abnormalities [28]. NAFLD is becoming increasingly common worldwide. NAFLD is defined by the presence of more than 5% fat content in the liver without any identifiable underlying cause [29]. It is the most common form of chronic liver disease, affecting approximately 25% of the world’s population, including an estimated 100 million people in the U.S. [30]. Periodontitis and NAFLD share numerous risk factors, and the progression of both conditions can be intensified by insulin resistance and elevated systemic inflammation, particularly in individuals with diabetes and obesity. In addition, overproduction of reactive oxygen species (ROS) has been consistent in the pathogenesis of periodontal disease and liver disorders [31].

Small intestinal bacterial overgrowth (SIBO) is a syndrome caused by an excessive number of bacteria in the small intestine. It is associated with a complex set of clinical manifestations, such as malabsorption, chronic diarrhea, macrocytic anemia, weight loss, or even protein-losing enteropathy or steatorrhea in more severe cases. It is estimated that up to 35% of the general population may suffer from bacterial overgrowth, and the prevalence may increase to 80–90% in patients with irritable bowel syndrome or chronic fatigue syndrome [19]. 

**Figure 1:**
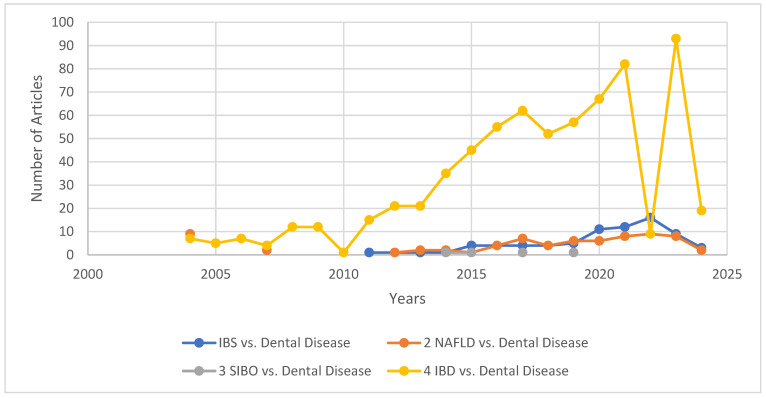
Chronology from 2004 to 2024 of the number of articles published using the keywords “dental” and “irritable bowel syndrome” (IBS), “non-alcoholic fatty liver disease” (NAFLD), “small intestinal bacterial overgrowth” (SIBO), and “inflammatory bowel disease” (IBD).

Interest has grown in exploring potential interactions between these two fields and is described in Figure 1 under the form of a timeline from 2004 to 2024 of the search count by using the keywords dental and the intestinal diseases referred to in this paper. We decided to make a statistic based on published articles that combine the issues of gastrointestinal diseases and dental disorders. In recent years, there has been a growing interest in a significant increase in interest related to the interactions between these two medical fields. From the search of current data, most papers were found on IBD in relation to dental disorders—681 articles. Followed by the interest in IBS and dental, with a total of 76 articles published over 20 years. A total of 71 papers were found on NAFLD and dental-related issues. A significantly smaller number of papers were found on the interaction between SIBO and dental; however, the publication of four publications does not mean that there is no interaction between these two diseases. Our search revealed that the number of searches using the keywords dental disease and gastrointestinal diseases increased significantly over the years following the 2004–2024 timeline, with the highest number of searches being recorded in 2023—110 articles, followed by the publication of 102 scientific papers in 2021. This is represented in Figure 1, along with the trend line intended to better illustrate the growing interest in the link between these medical fields.

An overview is provided of the current clinical and epidemiological evidence for the association between oral cavity disorders and liver and intestinal diseases, along with tracking their related mechanisms and discussing gaps to be filled by future research. 

## 2. Methodology

Studies were searched in the Pubmed, Web of Science database by using the following keywords: “dental/stomatological disorders”, “inflammatory bowel disease”, “small intestinal bacterial overgrowth”, “non-alcoholic fatty liver disease”, and “intestinal dysbiosis”, with cross-references of these keywords also counted in. In addition, we searched for unpublished trial data in clinicaltrials.gov (www.ClinicalTrials.gov/, accessed on 25 June 2024). This review article includes articles published from 1980 (when the first scientific mentions of the link between IBD and dental diseases appeared in a study in the journal Clinics in Gastroenterology, finding that patients diagnosed with IBD have a higher prevalence of oral lesions) to June 2024 in the main available databases. Only scientific articles published in English were selected for inclusion. Articles that did not meet the search requirements were excluded. 

## 3. Mechanistics

Several studies have investigated the correlation between oral disorders and a range of gastrointestinal diseases, including dyspepsia, gastroesophageal reflux, Helicobacter pylori infection, peptic ulcers, and transit disorders, as well as the potential risk of gastrointestinal malignancies [32,33]. Commonly observed oral manifestations include aphthous ulcers, a cobblestone appearance of the oral mucosa, pyostomatitis vegetans, gingivitis, periodontitis, angular cheilitis, and oral lichen planus [34]. Conditions of the oral cavity lead to tooth crowding, which results in aesthetic problems and functional discomfort for the patient. The major causes of tooth loss are periodontitis and dental caries. Periodontal diseases with destruction of the alveolar bone occur as a result of dysbiosis, often in patients with an impaired host response [35]. Despite affecting different bodily regions, these pathologies share common origins, and both demonstrate an exaggerated inflammatory reaction in susceptible individuals. It is therefore not surprising that recent epidemiological and clinical studies suggest an association between gastrointestinal diseases and periodontitis [22]. In the following paragraphs, we describe the correlation between periodontitis and the occurrence of dental caries in relation to gastrointestinal diseases. 

Oral health and the various factors related to it play a critical role in IBS pathology. There is emerging evidence pointing to and contributing to the abnormal presence of oral bacteria in the gut, which promotes triggering an inflammatory response by activating host immune defenses [30]. This evidence supports the notion of an imbalance in the “oral–intestinal” axis, characterized by a higher prevalence of periodontitis in patients with IBS and vice versa. It is conceivable that a disruption of the oral–gut–liver axis could initiate liver inflammation. One of the primary relationships between the gut microbiota and extraintestinal organs is the bidirectional gut–liver axis. This reciprocal relationship is facilitated by the portal venous system, which directly transports gut-derived products to the liver, and the liver reacts via the bile acid pathway and intestinal antibody production [36,37,38,39,40]. Consequently, the liver regulates the composition of gut bacteria, which in turn affects liver processes, particularly when the gut microbial balance is disrupted.

**Figure 2:**
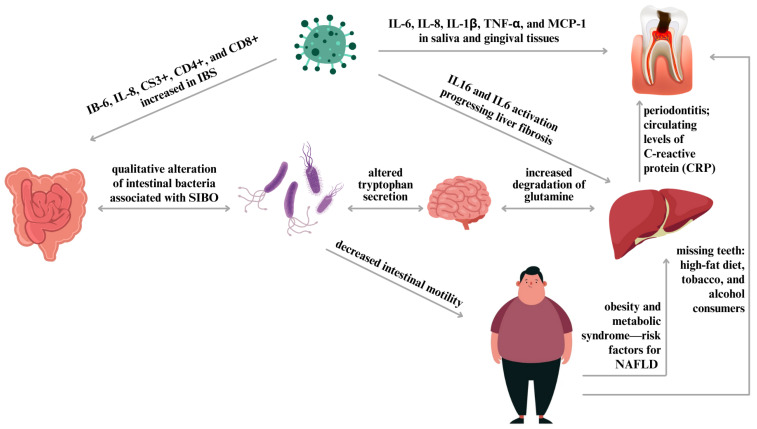
Interconnected pathways linking oral health, gut microbiome, and metabolic health. Processed schematic illustration summarizing the associations and coexisting etiologies of irritable bowel syndrome, non-alcoholic fatty liver disease, and bacterial overpopulation of the small intestine (modified after [28]).

Epidemiological research has linked the prevalence of missing or untreated teeth to cardiovascular diseases and diabetes, conditions that share common risk factors with NAFLD [41,42]. Multiple etiological factors intersect between IBS, NAFLD, and SIBO and oral cavity diseases; this has sparked interest in potential associations with obesity, gut microbiota composition, dietary habits, and immune-mediated processes, as illustrated in Figure 2. 

While the mechanisms of occurrence are different for each condition, there are some overlaps and complex interactions between oral health and gastrointestinal health. For example, chronic inflammation and dysbiosis in the oral cavity can impact gut health and vice versa, and a weakened immune system can make the body more susceptible to a variety of conditions, including periodontal and gastrointestinal diseases [43]. Analysis of patients with missing teeth revealed that periodontal disease risk factors often include systemic conditions such as diabetes, compromised immune function, stress, and obesity [44]. Another study examined an additional etiological mechanism, specifically investigating whether circulating serum C-reactive protein (CRP) levels and a weighted genetic CRP score, which represents inflammatory markers, may alter the relationship between periodontitis and NAFLD [39]. Data for this study were collected from 2481 participants enrolled in the baseline examination of the study of health in Pomerania, conducted between 1997 and 2001. Periodontitis was defined as the percentage of sites (0%, <30%, ≥30%) with pocket probing depth (PD) ≥ 4 mm, and NAFLD status was determined by liver ultrasound assessment.

A further study by Du et al. developed the theory that fibrogenic myofibroblasts in the liver exhibit dysregulated glutamine metabolism, illustrated in Figure 2 [45]. This hypothesis is particularly compelling in light of recent findings linking plasma glutamate levels to the severity of liver scarring in individuals with NAFLD. In a comprehensive study, Simón et al. established a clear link between fibrosis progression and elevated glutamine catabolism in multiple animal models of steatohepatitis, such as those exposed to a high-fat Western diet [46]. Glutamate is an excitatory neurotransmitter that may be involved in liver inflammation and NAFLD development. Changes in glutamate levels may influence insulin sensitivity and lipid metabolism in the liver, and lower serum glutamine levels were observed in aged mice diagnosed with advanced liver fibrosis. Another study reported [47] that glutamine serves as a primary energy source for intestinal epithelial cells; due to its reduced levels, glutamine might be a critical factor in the development of IBS symptoms. 

A recent study indicated that individuals exhibiting metabolic disorder symptoms had a diminished microbial capacity to convert tryptophan into compounds that activate the aryl hydrocarbon receptor (AhR). Tryptophan, a precursor to essential molecules like serotonin (5-HT) crucial for gut health, undergoes microbial transformation into various compounds, including indole and its derivatives. Some of these compounds bind to and activate the AhR. Activation of the AhR pathway correlates with reduced levels of glucagon-like peptide-1 (GLP-1) and interleukin-22 (IL-22), subsequently influencing intestinal permeability and lipopolysaccharide levels in functional gastrointestinal disorders. In addition, AhR agonists or Lactobacillus regulation may be a treatment available to reverse metabolic disorders. There are interconnections between the gastrointestinal diseases studied, as patients with NAFLD have a 13% higher risk of developing IBS. Obesity is frequently associated with IBS, while the link between NAFLD and IBS remains less evident. Research has indicated that SIBO occurs in 39% to 60% of NAFLD patients, although the study population was limited. However, more recent research identified SIBO in 8% of their NAFLD patient cohort without establishing a link between SIBO and a higher risk of developing fibrosing NAFLD. Although SIBO (small bowel bacterial overpopulation), NAFLD, and irritable bowel syndrome (IBS) are distinct conditions, there are certain aspects that may be common between them, and these are subjects of ongoing research [48]. 

### 3.1. Importance of Intestinal Dysbiosis in the Development of Oral and GI Diseases

Periodontitis and gastrointestinal disorders are interconnected, with chronic inflammation in either the mouth or intestines mutually affecting the other [49]. The 160 or so groups of microorganisms include *Firmicutes*, *Actinobacteria*, *Pseudomonadota*, *Fusobacteria*, and *Verrucomicrobia*. Firmicutes and Bacteroidetes bacteria phyla dominate, comprising 90% of the human gut microbiome [50]. The Firmicutes phylum encompasses more than 200 distinct bacterial genera. These include *Lactobacillus*, *Clostridium*, *Enterococcus*, and *Ruminococcus*. The Bacteroidetes phylum is primarily characterized by the *Bacteroides* and *Prevotella* genera. Both SIBO and IBS may involve imbalances of the intestinal flora. In SIBO, pathogenic bacteria invade the small intestine, and in IBS, microbial imbalances may play a role in digestive symptoms. Not only quantitative (SIBO) but also qualitative changes in intestinal bacteria (dysbiosis) have been reported among patients with IBS. The role of intestinal dysbiosis in the pathogenesis of IBS correlated with SIBO is also confirmed by the fact that symptoms in SIBO may occur following infectious gastroenteritis and in patients with diarrhea-predominant IBS. Several *Streptococcus* species are present in the oral cavity, including *Streptococcus mutans* and *Streptococcus sanguinis*. They can also be found in the small intestine, although they are not among the most common bacteria associated with IBS. *Prevotella* spp. is a genus of anaerobic gram-negative bacteria that can be found in the oral microbiota and may be associated with periodontitis. Some *Prevotella* species have also been identified in SIBO [51,52,53,54,55,56].

There is research suggesting that gut dysbiosis may be linked to NAFLD. The interaction between the gut microbiome and NAFLD is a growing area of research, and studies are focusing on how bacterial composition may affect liver inflammation and disease progression. In NAFLD, at the bacterial phylum level, a decrease in Bacteroidetes species is reported, while levels of Firmicutes, Proteobacteria, Enterobacteriaceae, Escherichia, and Citrobacter are increased. At the bacterial family level, *Enterobacteriaceae* was reported to be increased, while *Rikenellaceae*, *Anaerosporobacter*, *Coprococcus*, and *Ruminococcaceae* species are decreased. Different forms of the disease are characterized by the presence of different bacterial species. In NAFLD cases with steatosis alone, a higher level of Bacteroidetes was detected, while in NAFLD cases with fibrosis, a significantly higher proportion of Ruminococcus species was noted [57].

Individuals diagnosed with periodontitis could present with gut dysbiosis, although the precise origin and progression of this gut–oral microbial imbalance is yet to be determined. Similarly, liver dysfunction could aggravate ongoing pathological findings and clinical effects in the oral cavity. In a study by Arimatsu et al. [58], it was observed that in mice on a regular diet, oral administration of *Porphyromonas gingivalis* via gavage elevated the intestinal population of *Bacteroidetes* compared to *Firmicutes* and preceded a decrease in tight junction protein levels within the small intestine and an increase in proinflammatory cytokines (IL-6, IL-12p, IFN-γ, and IL-17c) in the large intestine, collectively contributing to intestinal barrier impairment. ROS that cause liver injury due to constant exposure to proinflammatory factors with subsequent production of proinflammatory cytokines also contribute to this process. Another experiment used intravenous delivery of sonicated *P. gingivalis* into mice’s high-fat diet and observed that pathogen lysates decreased the presence of *Ruminococcaceae* in the gut, a bacterial species that is absent in NAFLD [59]. Another research on animals suggested that the oral microbiota might also play a major role in the pathogenesis of NAFLD [60]; the study found that a specific strain of *Streptococcus mutans* could induce NAFLD in mice, *S. mutans* being generally considered the main pathogen responsible for the development of dental caries. Recent investigations have demonstrated that *S. mutans*, a primary causative agent of dental cavities, can trigger non-alcoholic steatohepatitis (NASH) in animal models [61]. Consequently, the link between tooth loss and NAFLD might indicate the harmful effects of *S. mutans* on both oral cavity health and overall wellness.

Cohort studies have evaluated the relationship between periodontal bacteria and the development of non-NAFLD. Alazawi and colleagues investigated the connection between gum disease and liver fibrosis in a population-based study that included 8153 participants and found a significant link between higher serum levels of *Streptococcus oralis* antibodies and hepatic steatosis [62]. Another bacterium that is correlated with the progression of liver fibrosis is *P. gingivalis*. In their study, Nakahara et al. detected serum immunoglobulin levels targeting *P. gingivalis* fimA type 4 in biopsy samples from patients diagnosed with NAFLD [63]. It is not yet known whether the pathogen *P. gingivalis* could migrate from the oral cavity to the liver and whether it may aggravate the pre-existing gut dysbiosis found in patients with NAFLD [28].

Several studies that have led to disease in vivo in animal models (rats, mice, and rabbits) assessed the effect of major periodontal bacteria, particularly *P. gingivalis*, on liver tissues employing various administration methods, including oral delivery and injections of live bacteria or bacterial extracts into the dental pulp, peritoneal cavity, bloodstream, and gingival tissues, have confirmed that specific periodontal bacteria can directly harm the liver [64,65,66]. This species of bacteria found in the oral flora is linked to the occurrence of intestinal tumors. Extensive genomic research has identified specific oral bacteria associated with colorectal cancer, including *P. gingivalis* [67]. Bacteria can also target the immune system. The gastrointestinal tract is the primary site of interaction between the immune system and external factors. The role of *P. gingivalis* in the etiology of autoimmune dysfunctions, which are complex trait disorders, can be emphasized. *P. gingivalis* is recognized as the principal microbe causing periodontitis, and elevated levels of antibodies against *P. gingivalis* have been identified in the blood of individuals at heightened risk for developing rheumatoid arthritis (RA) as well as in those with RA. Importantly, periodontitis caused by *P. gingivalis* and *P. nigrescens* can worsen the progression of experimentally induced arthritis in mice, intensifying the severity of the joint inflammation [68].

Under these circumstances, the interplay between oral and gut microbial communities is intricate, constantly changing, and influenced by various factors, as both ecosystems exist within the multifaceted environment of the digestive system. Under physiological conditions, they can maintain a regulated balance throughout life, but disruptions in this interaction due to an imbalance of host microbes may initiate inflammatory cascades in various tissues. The symbiotic relationship between *P. gingivalis* and *Veillonella rogosae* bacteria can make a symbiotic relationship between them, and the host can be established in the oral cavity, unlike the small intestine, where their presence can disrupt the ecosystem and potentially lead to intestinal disease [69]. 

**Figure 3:**
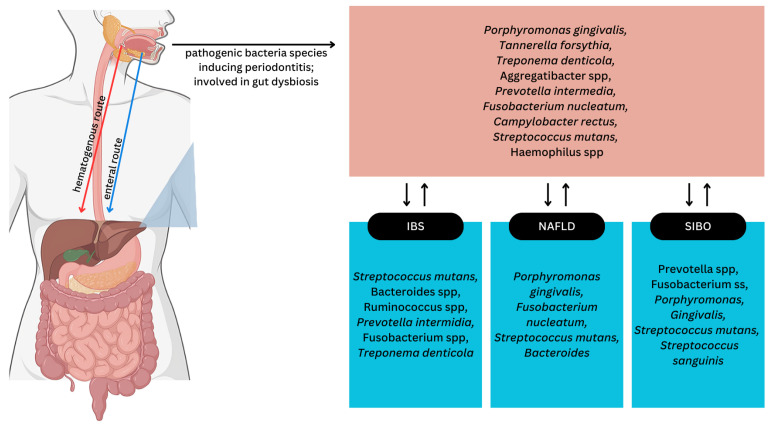
Oral bacteria in the context of periodontal dysbiosis could also invade the gut, causing adverse effects on the gut microbiome and affecting liver and intestinal function.

Some bacteria can be spread from one place to another, where they can cause systemic disease. Two pathways have been proposed by which pathogenic bacteria would migrate from the oral cavity to the intestinal mucosa: the hematogenous pathway and the enteral pathway [47], illustrated in Figure 3. Research has demonstrated that everyday dental actions like vigorous chewing and brushing, as well as dental treatments such as orthodontic work and extraction, can cause physical damage to the mouth, allowing oral bacteria to enter the bloodstream. This was supported by the study by Horliana et al., which found *P. gingivalis* periodontal pathogens in the bloodstream of periodontitis patients [69]. An alternative pathway for dissemination of oral bacteria is by enteral spread. After disruption of the epithelium, oral microorganisms ingested may engage with gut-associated immune cells, stimulating the production of proinflammatory cytokines such as IL-17, IL1β, and IFN-γ. Furthermore, Th17 cells reactive to oral pathogens that arise de novo in the oral cavity can translocate to the inflamed gut, where they are activated by ectopic oral colonizations. Oral pathogens later contribute to intestinal inflammation. Consequently, periodontitis can worsen intestinal inflammation by introducing inflammation-inducing oral bacteria and harmful immune cells into the gut. Typically, oral bacteria struggle to establish themselves in the healthy gut due to protective barriers within the digestive system. However, when the gut’s microbial balance is disrupted, oral bacteria can more readily colonize the intestines. An example of this may be bacterial translocation by *Klebsiella* spp., which is present in the saliva of IBS patients. This bacterium is resistant to several antibiotics, including vancomycin and penicillin, so it can translocate into the intestinal mucosa. Individuals with impaired stomach function due to insufficient stomach acid have been found to harbor oral bacteria in their intestines (e.g., *Streptococcus* spp., *Veillonella* spp., *Haemophilus* spp.; Figure 3). Remarkably, some oral bacteria, including *P. gingivalis*, can survive the stomach’s acidic conditions and subsequently penetrate the stomach lining. Therefore, while stomach acid can hinder the passage of oral bacteria into the intestines, its effectiveness is reduced against bacteria resistant to acidic conditions [70,71,72,73,74,75].

The intestinal microbiota is essential for maintaining gut health, processing nutrients, and developing immune tolerance. Microbiome dysregulation has been proven to play a part in the development of both IBS and NAFLD [76]. The gut’s bacterial community inhabits a complex and varied environment. It is crucial to differentiate between the gut bacteria residing within the intestinal tract, those attached to the intestinal lining, and those present in stool samples, as these populations can differ significantly. In subjects with IBS, fecal microbiota studies found an increase in facultative anaerobic bacteria (such as *Escherichia coli* and *Clostridium*) and a decrease in lactobacilli and bifidobacteria [76]. Other studies show a correlation between changes in the *Firmicutes* and *Bacteroidetes* ratio in IBS patients and similarly observe a reduction in lactobacilli and bifidobacteria species. Additionally, periodontitis can lead to bacteremia, with *P. gingivalis*, *Fusobacterium nucleatum*, *Treponema denticola*, and *Prevotella intermedia* being able to circumvent immune defenses and replicate within immune cells [69] and possibly aggravating IBS development. Research in animals has shown that oral antibiotic treatment imbalances the gut bacteria, diminishes the number of lactobacilli, and reduces populations of bacteroides and enterococci, ultimately influencing pain sensitivity and gut-muscle responses [76]. *Lacticaseibacillus rhamnosus* also has a protective role in pain prevention demonstrated in animal models. Multiple studies propose that an imbalance of oral bacteria, coupled with subsequent gut inflammation, may contribute to oral complications in IBD [77,78]. Excessive levels of specific oral bacteria, such as *Streptococcus*, *Prevotella*, *Veillonella*, and *Haemophilus*, in the mouth are connected to inflammatory reactions caused by decreased salivary lysozyme and elevated IL-1β levels, which may be related to an imbalance of gut bacteria (Figure 3) [79]. Table 2 summarizes the interconnection of several oral microorganisms mentioned above according to the digestive system disease.

### 3.2. Common Immunoinflammatory Responses

An inflammatory disruption caused by one illness can either directly impact the progression of the other or may occur independently in a susceptible individual with a shared immune system dysfunction. This causes the development of chronic general disorders [41]. Based on the findings from the reviewed epidemiological and clinical research, it is conceivable that an imbalance in the oral–gut–liver relationship could initiate additional liver inflammation. Inflammation is a common feature of gastrointestinal diseases and can also affect the oral cavity. In both the progression of IBS and periodontal disease, the condition is driven primarily by inflammatory processes involving tissue-destructive cytokines. In IBS, inflammation may be caused by the immune system’s response to bacteria in the small intestine. In NAFLD, liver inflammation is a key aspect of the disease [41].

During periodontal disease, circulating neutrophils Nφs and Mφs, cells constituting the primary liver cell population, obtained from the blood of periodontitis sufferers, triggered a hyperinflammatory state defined by persistently high production of inflammatory cytokines (e.g., IL-1, IL-6, TNF, CXCL8). In a similar manner, Mφs derived from patients affected by NAFLD presented a hyperinflammatory state and diminished phagocytic capacity [80].

Chronic inflammatory responses are essential for the advancement of NAFLD, and considerable strides have been made in comprehending the significance of inflammation [81]. Natural killer and natural killer T cells contribute to inflammation by producing inflammatory signaling molecules and ROS. It has been shown that the tumor necrosis factor TNF-α, along with other cytokines and growth factors, may play a role in the development of NAFLD in both animals and humans. The combined action of TNF-α and IL-6 induces leptin production, thereby activating neutrophils and the immediate immune system. Moreover, adaptive immunity fuels the progression of NAFLD as the accumulation of B cells and CD4+ and CD8+ T cells within the liver intensifies tissue damage and inflammation [82]. B cells promote liver fibrosis through the activation within hepatic stellate cells and macrophages. CD4+ T cells transform into T helper 17 cells, generating interleukin-17, implicated in the progression of NAFLD. Equilibrium of adaptive immune cells compartment in the liver can shift from a pro-resolving to a proinflammatory status, leading to disease and fibrosis. Several studies highlight the impact of cytokine levels in both gastrointestinal and oral regions. Increased levels of inflammatory markers, including IL-6, IL-8, IL-1β, TNF-α, and MCP-1, were detected in both oral fluids and gingival tissue from IBS sufferers relative to those in remission [35].

IBS has been described as a chronic inflammatory condition of similar degree but lower intensity. The body’s initial immune defense is implicated in IBS, with elevated mast cell counts observed in the gastrointestinal tract of certain patients. Additionally, the acquired immune system plays a role, as evidenced by increased numbers of CD3+, CD4+, and CD8+ T cells in both the intestines and bloodstream. Notably, a rise in IL-6 and IL-8 with a reduction in anti-inflammatory cytokines was observed in the serum of IBS patients. TLRs are considered essential in this process, and IL-6 along with other cytokines operate via this mechanism. TNF-α can act on the nervous system and cause hypersensitivity, gastric hypomotility, and nausea. The advancement of both IBS and periodontal disease is primarily driven by immune and inflammatory responses mediated by cytokines that cause tissue damage. Menegat et al. noticed that significant upregulation of cytokines IL-17A, IL-17F, IL-22, IL-25, IL-33, IL-10, and IFN-γ was observed in gingiva relative to intestinal mucosa within the IBS patient population exhibiting periodontal disease. Specifically, *Enterobacteriaceae* such as *Klebsiella* isolated from the oral cavity trigger IL-1β production by macrophages, enhancing intestinal secretion of intestinal inflammation by mediating IL-1 signaling. Interestingly, Enterobacteriaceae bacteria extracted from the intestines do not induce comparable IL-1β release, implying a selective reaction to oral microorganisms. It would follow that macrophages play a key role in the immunological response and are involved in both periodontitis and IBS [83,84].

SIBO is known to be associated with increased levels of IL-8. This phenomenon is explained by the fact that bacteria deconjugate bile salts present in the gut. These deconjugated bile salts can increase fluid output from the diarrhea resulting from colon dysfunction. Subsequently, free bile acids, which are toxic to the intestinal mucosa, can cause inflammation of the mucosa and the release of pro-inflammatory cytokines. Several studies highlight the influence of cytokine production in both the gastrointestinal and oral regions. Genetic factors play a role in determining the host response to SIBO, as supported by a case–control investigation of 209 IBS patients and 273 healthy individuals, underproducing genotypes of the interleukin-1 (IL-1) receptor antagonist gene (were associated with IBS) [85]. Furthermore, patients with IBS had higher levels of IL-1 α and β than those without SIBO. A separate study revealed elevated concentrations of proinflammatory cytokines, including IL-6 and TNF- α, in individuals with IBS in comparison to healthy participants in the research. In addition, SIBO is correlated with elevated levels of circulating endotoxins, inflammatory signaling molecules, and endogenous ethanol production [86].

Cytokines, including IL-1A, IL-1B, IL-10, and IL-6, released during gastrointestinal disturbances, are pivotal in driving the inflammatory response in periodontal disease [87]. These intermediaries influence the stimulation, proliferation, and development of B cells, the main cells involved in the severe manifestations of periodontitis; these results show the interconnections present between the different component parts of the digestive system [88].

### 3.3. The Interaction of Gut Motility Disorders with Oral Conditions

Observed changes in healthy gut microbiota concentrations can lead to systemic changes such as intestinal motility disorders, hypertension, and atherosclerosis [89]. Both SIBO and IBS are associated with gut motility disorders. In SIBO, excess bacteria can affect normal small bowel movements. IBS is characterized by changes in colon motility. Abnormal bowel motility results from a number of processes. First, bile acids are passively absorbed and activated with Takeda G protein-coupled receptor 5 (TGR5) by enteric neurons. Second, the effect of bile acid-induced colon dysfunction may result from microbial dysbiosis [50]. A disrupted gut flora damages intestinal tight junctions and consequently increases mucosal permeability, which allows bacterial metabolites and microbe-associated molecular patterns/damage-associated molecular patterns (MAMPs)/(DAMPs) to reach the liver via the portal venous system and in several cases promotes endotoxemia [28]. Subsequently, these molecules directly engage hepatocytes, Kupffer cells, and hepatic stellate cells (HSCs), stimulating pattern recognition receptors such as TLRs and NLRs, which in turn initiate inflammatory responses [90].

Research suggests that obesity decreases intestinal movement, potentially contributing to SIBO through stagnation. It is hypothesized that this may also weaken the intestinal barrier, allowing bacteria to migrate and expand the gut–liver connection. In addition, changes in the gut–pharyngeal axis may be a consequence of increased intestinal permeability [46]. There is a hypothesis explaining the role of the immune system and inflammation in disrupting intestinal motility. In an experiment on mice infected with Trichinella spiralis, it was observed that they developed intestinal muscle hypercontractility, but these effects disappear in CD4-deficient animal models [91].

The significant effect of the gut microbiome on systemic well-being has gained substantial attention lately [92]. Oral conditions involving tooth loss or other chewing and swallowing problems can lead to difficulties in eating properly. It can lead to nutrient deficiencies and affect gut motility by interfering with the normal absorption of nutrients needed for a well-functioning gastrointestinal tract. Patients with multiple cavities or missing teeth have a shorter chewing time and experience chewing difficulty. A study by Ledari et al. (2012) found that chewing gum mastication was used clinically to treat postoperative ileus by stimulating bowel function, reducing flatulence, and promoting defecation [50]. At the same time, edentulous individuals’ food consumption can differ from dental persons. Thus, edentulous people prefer semi-solid or solid foods. In a study of a group of mice, it was observed that powdered food caused constipation-like side effects due to mild colitis, which were improved using neutrophil-depleting drugs and neutrophil elastase inhibitors. Lastly, the decreased colon activity of mice consuming a powdered diet was significantly stimulated by two hours of enforced chewing. Therefore, proper chewing may be crucial for cultivating a healthy gut ecosystem by fostering beneficial interactions between intestinal bacteria and the body’s immune response [93]. 

### 3.4. Lifestyle Factors and Diet

Dietary habits and lifestyle factors, as well as high-sugar diets or processed foods, can play a role in the creation of both oral disorders and gastrointestinal problems. These habits can influence the oral and gastrointestinal microbiome, contributing to microbial imbalances. Several factors cause gut dysbiosis, e.g., diets and sweetening agents can increase opportunistic gut colonization by oral bacteria. These factors can influence the oral and gastrointestinal microbiome, contributing to microbial imbalances. High-sugar diets and processed foods contribute to both oral disorders (e.g., dental caries, periodontal disease) and gastrointestinal problems (e.g., gut dysbiosis). These diets can promote microbial imbalances that negatively impact health. Balanced nutrition is essential for maintaining optimal health. Diets high in fiber, vitamins, and nutrients support both oral and gut health, while poor nutrition can exacerbate health issues. 

Obesity is intrinsically linked to non-alcoholic fatty liver disease (NAFLD), diabetes, and metabolic syndrome. It can reduce intestinal motility and contribute to gut dysbiosis, leading to conditions such as small intestinal bacterial overgrowth (SIBO). Weight loss via diet and exercise diminishes liver fat and scarring, enhancing general well-being.

Both smoking and excessive alcohol consumption are detrimental to oral health, contributing to tooth loss and oral cancers. These habits are also linked to the development and progression of NAFLD, emphasizing the importance of addressing these lifestyle factors in both oral and systemic health.

Proper dental care and oral hygiene are essential for preventing oral diseases that can impact systemic health. Poor oral health, including conditions like edentulism (tooth loss), can lead to dietary changes that negatively affect overall health. Individuals with poor dentition often select foods with a smoother texture that are high in saturated fat and cholesterol, which can result in metabolic disturbances such as NAFLD [50].

Regular physical activity supports overall health, including digestive health. Exercise can enhance gut motility and reduce the risk of obesity-related complications.

Adequate sleep and effective stress management are important for maintaining a healthy gut–brain axis. Chronic stress and sleep deprivation can exacerbate both gastrointestinal and oral health problems.

Dental health care and balanced nutrition should be approached holistically to ensure optimal whole-body health [38].

NAFLD is closely associated with obesity, diabetes, and metabolic disorders. The occurrence of NAFLD can reach as high as 95% in obese populations [94]. Excess adipose tissue that depletes peripheral storage capacity, leading to deposition in the liver and increased insulin resistance, is considered to be the main factor in the pathogenesis of NAFLD, shown in Figure 2. Dietary and exercise-induced weight loss can diminish fatty liver and scarring. Additionally, a study by Lassailly et al. involving 109 obese individuals demonstrated that bariatric surgery eliminated NASH within twelve months [95].

Obesity or weight loss may also be present in oral disorders [96]. A US study [97] showed that people with partial or total edentulousness tend to opt for foods that require less chewing, such as those high in saturated fat and cholesterol, for foods rich in carotenes, fiber, and vitamin C. As a result, people with dental problems are inclined to have lower fiber intake and higher carbohydrate intake. This dietary pattern could partially elucidate the association between NAFLD and decreased tooth count. The detrimental effects on chewing ability and diet quality due to missing teeth may contribute to this link. Another factor that intersects with both diet and health-damaging behavioral habits is smoking and alcohol consumption, which are associated with a higher risk of both tooth loss and NAFLD. Smoking and excessive alcohol consumption are known contributors to tooth loss due to their detrimental effects on oral health. Additionally, these habits are also linked to the initiation and progression of NAFLD, further underscoring the importance of addressing lifestyle factors in oral and systemic health.

The relationship between IBS and obesity is not entirely clear. Aro et al. discovered a strong link between obesity and IBS symptoms such as abdominal pain and diarrhea when using the Abdominal Symptoms Questionnaire. However, these have not been confirmed in several other studies [98]. Some types of food seem to worsen IBS symptoms and play a role in the development of NAFLD. High fructose corn syrup (HFCS) is a disaccharide that is commonly used in artificial sweeteners, processed, canned, and bakery products around the world. Excessive carbohydrate consumption may contribute to symptoms of NAFLD. HFCS has been demonstrated to trigger IBS symptoms manifestations by elevating osmotic tension and bacterial fermentation, leading to gas, bloating, and abdominal pain [28]. HFCS has also been shown to suppress the insulin signaling pathway, which could contribute to the development of NAFLD. Fructose consumption is associated with heightened intestinal permeability, potentially leading to NAFLD and IBS, through the mechanisms already discussed [41]. More research is certainly needed on the dietary implications for NAFLD and IBS. Many IBS patients notice that “healthy” foods, such as fruits and vegetables, can worsen their symptoms, leading some to adopt an “unhealthier” diet, which can lead to weight gain. There is evidence that a low FODMAP (fermentable oligosaccharides, disaccharides, monosaccharides, and polyols) diet, which excludes some fruits and vegetables, ameliorates IBS symptoms, but research by other authors suggests that many IBS patients report sensitivity to foods with low content in FODMAP [99]. 

## 4. Limitations and Future Directions

This review encounters several limitations that must be acknowledged. First, the restriction to articles published in English may have resulted in the omission of relevant studies conducted in other languages, which could provide additional insights or differing perspectives on the interconnections between oral cavity diseases and “modern” digestive diseases. Future reviews should consider including major studies available in other languages, possibly using translation services to ensure a more comprehensive coverage of global research.

Secondly, the methodology employed in this review does not include a quantitative analysis of the data because of the varied characteristics of the studies reviewed. This limits our ability to perform a meta-analysis that might offer more definitive conclusions regarding the strength and nature of the associations discussed. Future research could aim to standardize the data collection methods in this field to allow quantitative synthesis of the data.

Additionally, the possibility for publication bias exists, as studies with non-significant findings are less likely to be published. This review did not include unpublished studies or gray literature, which might contain important data relevant to our understanding of these diseases. Researchers should consider including such sources in future reviews to mitigate the impact of publication bias.

In terms of future directions, there is a significant need for longitudinal studies that can more conclusively determine causative relationships between oral and digestive diseases. Moreover, experimental studies that manipulate the oral microbiota to observe direct effects on digestive health could provide compelling evidence to support the hypotheses proposed by observational studies. Furthermore, interdisciplinary research involving both dental and gastroenterological specialists could enhance the comprehension of the intricate relationships among oral health and digestive diseases, leading to more integrated approaches in both diagnostics and treatment. 

## 5. Conclusions

This research offers a significant understanding of the complex interactions between oral cavity disorders and modern digestive diseases such as IBS, NAFLD, and SIBO. By exploring the altering of the gut microbiome through various interventions, we have highlighted potential therapeutic benefits and underlying mechanisms that could enhance patient outcomes. Weight management, nutritional adjustment, lifestyle interventions, and therapy for underlying metabolic syndrome remain the mainstays of therapy once the diagnosis is established. While these regimens lead to promising results, they require considerable effort from patients. The current state of knowledge about oral cavity disorders and modern digestive diseases has evolved significantly, revealing complex interactions between these systems. A deeper understanding of these connections offers new insights into diagnosing and treating these medical conditions, ultimately enhancing patients’ life quality. The most significant findings of our study include the identification of key microbial alterations associated with these diseases and the promising results from microbiota-based therapies such as prebiotics, probiotics, synbiotics, and FMT. These findings underscore the importance of a holistic approach in diagnosing and treating these interconnected conditions, highlighting the necessity for further collaborative research to optimize the performance and integrated treatment strategies.

## Figures and Tables

**Table 1:** Clinical trials involving microbiota modulation and diseases of the oral–gut axis.

Trial Name	Disease Focus	Study Description	Status	Identifier Number (www.ClinicalTrials.gov)
Fecal microbiota transplantation in patients with IBS	IBS	Post-infectious IBS patients with moderate to severe symptoms.	Ongoing	NCT03333291
Effects of continuous treatment with rifaximin and probiotics on the gut microbiota of patients with IBS-D	IBS	Effects of repeated rifaximin and sequential rifaximin–probiotic treatment on symptoms and quality of life. Next-generation sequencing and qPCR assess the impact of medications on the intestinal flora.	Ongoing	NCT04074421
The effect of consecutive fecal microbiota transplantation on non-alcoholic fatty liver disease	NAFLD	The impact of consecutive FMT on liver fat accumulation, as measured by magnetic resonance imaging (MRI).	Ongoing	NCT04465032
Evaluate the effect of supplementation of probiotics on liver changes (histological and enzymatic), lipid profile, and gut microbiota of patients with non-alcoholic steatohepatitis (NASH).	NAFLD	Impact of probiotic supplementation on liver tissue and enzyme alterations, blood lipid levels, and intestinal microbial composition in individuals with non-alcoholic steatohepatitis (NASH).	Ongoing	NCT02764047
The safety and efficacy of a probiotic intervention on SIBO and related gastrointestinal symptoms	SIBO	Impact of a probiotic formulation on gastrointestinal symptoms and gut and small intestine microbiota in SIBO. Two doses of probiotics are evaluated against placebo.	Ongoing	NCT06317441
Clinical study of fecal microbiota transplantation in the treatment of small intestinal bacterial overgrowth	SIBO	Overall efficacy and safety of FMT in treating SIBO.	Ongoing	NCT06162702

**Table 2:** Correlation between oral microbiota and digestive system diseases: a comprehensive review of pathogenic interactions.

Oral Flora	Digestive System Disease	Relationship	Reference
*Streptococcus mutans*	NAFLD, NASH	Can induce NAFLD and NASH in a mouse model, being generally considered the main pathogen responsible for the development of dental caries.	[61]
*Porphyromonas gingivalis*	NAFLD, Colorectal cancer	Increases *Bacteroidetes*, affects the intestinal barrier, and induces liver damage. Association with the development of colorectal cancer due to its inflammatory effects.	[59,67]
*Veillonella* spp.	IBS	Linked to periodontitis and IBS. Cause an imbalance and possibly lead to the development of intestinal disease.	[75]
*Treponema denticola*	IBS	Can evade immune surveillance, exacerbates IBS.	[75]
*Prevotella intermedia*	IBS	Linked to periodontitis and IBS.	[75]
*Fusobacterium nucleatum*	IBS	Can evade immune surveillance, exacerbates IBS.	[75]
*Haemophilus* spp.	IBS	Found in the gut of patients with gastric dysfunction related to achlorhydria.	[72]
*Prevotella* spp.	SIBO, IBS	Found in the oral microbiota, associated with periodontitis and SIBO. Some *Prevotella* species have also been identified in SIBO.	[55]
